# Is there an association between anxiety and depression prior to and during pregnancy and gestational diabetes? An analysis of the Born in Bradford cohort

**DOI:** 10.1016/j.jad.2020.07.019

**Published:** 2020-11-01

**Authors:** Claire A Wilson, Gillian Santorelli, Josie Dickerson, Khalida Ismail, Rebecca M Reynolds, Emily Simonoff, Louise M Howard

**Affiliations:** a Section of Women's Mental Health, King's College London and South London and Maudsley NHS Foundation Trust, PO31 King's College London, De Crespigny Park, London, SE5 8AF, UK; b Born in Bradford, Bradford Teaching Hospitals NHS Foundation Trust, Bradford, UK; c Department of Psychological Medicine, King's College London and South London and Maudsley NHS Foundation Trust, UK; d Centre for Cardiovascular Science, University of Edinburgh, UK; e Department of Child and Adolescent Psychiatry, King's College London and South London and Maudsley NHS Foundation Trust, UK

**Keywords:** Diabetes, Gestational, Perinatal care, Common mental disorders, Born in Bradford

## Abstract

**Background:**

anxiety and depression are common in women with gestational diabetes but it is not clear whether they are more likely to precede the onset of gestational diabetes or to co-occur with it. Our aims were to compare the strength of association between common mental disorders of anxiety and depression (i) before pregnancy and (ii) during pregnancy in women with and without gestational diabetes.

**Methods:**

the sample comprised 12,239 women with 13,539 pregnancies from the UK's Born in Bradford cohort. Gestational diabetes was diagnosed by oral glucose tolerance test (OGTT). Indicators of common mental disorders were obtained from linked primary care records. Multivariable robust Poisson and logistic regression were employed. Multiple imputation by chained equations was implemented to handle missing data. Models were adjusted for maternal age, ethnicity, education and obstetric complications. Analyses of common mental disorders during pregnancy were additionally adjusted for maternal smoking, pre-pregnancy BMI, multiple pregnancy and common mental disorders prior to pregnancy.

**Results:**

there was no evidence for an association between common mental disorders prior to pregnancy and gestational diabetes (adjusted RR 0.96; 95% CI 0.80,1.15) or between gestational diabetes and common mental disorders during pregnancy (adjusted OR 0.91; 95% CI 0.73,1.12).

**Limitations:**

high levels of deprivation and multi-ethnic composition of the cohort may limit generalisability of these findings to other populations.

**Conclusions:**

routine primary care records did not identify an increased risk of gestational diabetes in women with common mental disorders prior to pregnancy or of gestational diabetes in women with common mental disorders during pregnancy.

## Highlights

• No association between gestational diabetes and common mental disorders prior to and during pregnancy.
• One of the largest cohort studies in multi-ethnic UK population of 12,239 women with 13,539 pregnancies.
• No increase in risk observed with increasing levels of hyperglycaemia used as a continuous measure.
• Reassuring findings for women with GDM and/or CMD.

## 1 Introduction

Gestational diabetes mellitus (GDM) is diabetes which occurs for the first time during pregnancy and has a global prevalence of between five and 10 percent. It is associated with negative outcomes for mother and baby, including obstetric complications (Kampmann et al., 2015) and adverse metabolic and neuro-behavioural outcomes in offspring (Ornoy, 2005).

The common mental disorders (CMD) of anxiety and depression are also common morbidities to affect women both during and prior to pregnancy; they too are associated with adverse obstetric and longer-term offspring outcomes (Howard et al., 2014). There is an increasing awareness of the physical-mental health interface and there is now a body of research supporting a bidirectional relationship between depression and Type 2 diabetes (Moulton et al., 2015). However, the physical-mental health interface in pregnancy, in particular the association between GDM and CMD has been relatively less investigated. Given that there is the pathophysiology of insulin resistance common to both GDM and Type 2 diabetes, we hypothesised that there may be an association between GDM and CMD. Potential mechanisms for this association between GDM and CMD include insulin resistance secondary to placental hormone secretion, inflammation and shared socioenvironmental risk factors (Osborne and Monk, 2013).

In a recent systematic review and meta-analysis, we found an increased risk of depression in women with GDM, with the greatest risk during pregnancy, around the time of GDM diagnosis (Wilson et al., 2020). None of the studies were of a UK population and despite an increasing awareness of the influence of preconception mental health on pregnancy outcomes (Patton et al., 2018; Wilson et al., 2018) there have been few studies investigating the risk of GDM in women with preconception mental disorders. A small number of North American cohorts have found an increased risk of GDM in women with anxiety and depression prior to pregnancy (Beka et al., 2018; Bowers et al., 2013; Clark et al., 2019) but it is not clear the extent to which these findings generalise to a UK population.

Thus the aim of this study was to investigate the relationship between GDM and CMD both prior to and during pregnancy in the UK's Born in Bradford cohort. We hypothesised that there would be an association with GDM in women with CMD prior to pregnancy and also during pregnancy.

## 2 Methods

### 2.1 Sample

Born in Bradford (BiB) is a prospective longitudinal cohort of 12,450 women with 13,758 pregnancies in Bradford. Bradford is a city in the north of England and one of the most economically deprived urban areas of the UK, with 60% of babies born in Bradford being born into the poorest 20% of the English and Welsh population according to Index of Multiple Deprivation. Its ethnic makeup is predominantly bi-ethnic: Pakistani and White British.

**Fig. 1:**
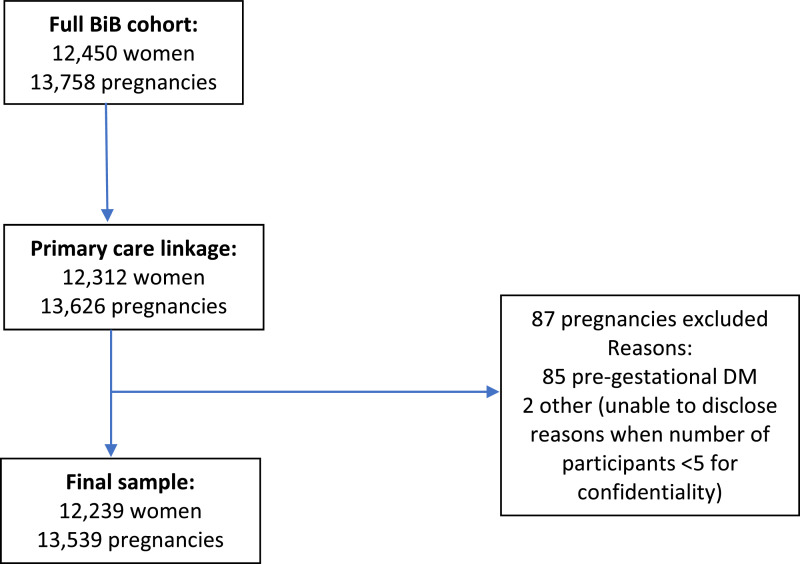
How the sample was obtained.

Pregnant women were recruited to BiB between 2007 and 2010 (Wright et al., 2013), when attending a routine appointment for a two hour 75g oral glucose tolerance test: OGTT; this is currently recommended by the UK's National Institute for Health and Care Excellence (NICE) as gold standard for diagnosing GDM (National Institute for Health and Care Excellence (NICE), 2015). It is offered to all women in Bradford and this is usually between 26 and 28 weeks gestation. Consent was obtained for record linkage to maternity and primary care records via SystmOne: a clinical computer system used by almost all general practices in Bradford and which provides primary care data on diagnoses and prescriptions. Women without linkage to primary care records or with pre-gestational diabetes (Types 1 and 2) were excluded from the analyses (see Fig 1). This gave a final sample of 12,239 women with 13,539 pregnancies.

### 2.2 Measures

#### 2.2.1 GDM

GDM was diagnosed according to modified World Health Organisation (WHO) criteria (either fasting glucose ≥6·1 mmol/l or two hour post-load glucose ≥7·8 mmol/l) (Farrar et al., 2015). The primary outcome was a binary variable of GDM or no GDM. Fasting glucose results were also analysed as a continuous variable in sensitivity analyses.

#### 2.2.2 Common mental disorders (CMD)

As an indicator of CMD, Read codes (CTV version 3) for diagnosis and/or treatment of CMD were used alongside medication prescriptions as per previously published methods (Prady et al., 2016). These relate to ICD-10 diagnostic groups F32 and F33 depressive episodes and recurrent depressive disorders and F41 anxiety disorders (WHO, 1990). ‘Preconception’ CMD as a binary variable was defined as any indicator of CMD that was dated from the woman's birth to the date of conception (estimated from date of last menstrual period and/or booking scan) of the woman's first ever pregnancy. The binary variable of ‘antenatal’ CMD was defined as any indicator appearing between the date of conception and the date of delivery for each pregnancy in the BiB cohort. Further information on the codes used is provided in supplementary material (S1).

#### 2.2.3 Confounders

There were a number of variables identified as potential confounders due to their association with both GDM and CMD but not on the causal pathway from exposure to outcome (VanderWeele, 2019). These included maternal age (Anna et al., 2008; McManus et al., 2016) at the time of the OGTT and other obstetric complications that usually occur around the same time as GDM (as a binary variable of pre-eclampsia and/or gestational hypertension) (Alder et al., 2007; Kim et al., 2005; Xiong et al., 2001). Also included were the sociodemographic characteristics of maternal ethnicity (as a three category variable of Pakistani, White British or Other) and socioeconomic status (SES) (Anna et al., 2008; Bhui et al., 2001; Howard et al., 2014); rather than using index of multiple deprivation (Department for Communities and Local Government, 2015) to assign SES, a five category variable of maternal education was used a proxy, as the high levels of deprivation in BiB result in a highly skewed distribution of participants across deprivation categories. Analyses of antenatal CMD additionally controlled for preconception CMD, any tobacco smoking during pregnancy (Bar-Zeev et al., 2020; Tong et al., 2016), singleton versus multiple pregnancy (Ben‐Haroush et al., 2004; Ross et al., 2011) and the continuous variable of BMI (body mass index) at pregnancy booking as a measure of pre-pregnancy BMI (Molyneaux et al., 2014).

### 2.3 Statistical analysis

Data were analysed using Stata version 15 (StataCorp, 2007). Multivariable robust Poisson and logistic regression were employed to examine the associations between GDM with preconception CMD and antenatal CMD respectively, within a generalised estimating equation framework using the robust sandwich estimator for standard errors to account for multiple pregnancy clustering. Two analyses were performed: unadjusted and adjusted for the confounders described above, to produce risk ratios (RR) for the Poisson regression models and odds ratios (OR) for the logistic regression models. An interaction term for ethnicity was also included in the models.

Multiple imputation by chained equations was implemented to handle missing data (White et al., 2011). 35 imputations were used according to the proportion of participants with any missing data (Bodner, 2008). All analysis variables were included in the imputation model. Estimates were obtained by pooling results using Rubin's rules (Rubin, 1987). Complete case analyses were also conducted (see supplementary material).

#### 2.3.1 Subgroup analyses

Analyses were stratified into two groups of Pakistani and White British ethnicity, using the above analysis but with the removal of ethnicity as a covariate. As these were the most prevalent ethnicities, further categories of ethnicity such as ‘Other’ would have been too heterogeneous.

#### 2.3.2 Sensitivity analyses

The following sensitivity analyses were performed on the full sample in addition to the primary analysis:

(1) The continuous variable of fasting glucose was used instead of the binary GDM variable used in the primary analyses using linear regression.
(2) The primary GDM variable was also re-classified using fasting and two hour post-load glucose from OGTT. Given the widespread debate surrounding GDM diagnosis (Koning et al., 2018), two different sets of diagnostic criteria were applied: 1) current NICE criteria (either fasting ≥5.6 mmol/l or two hour post-load glucose ≥7·8 mmol/l) (National Institute for Health and Care Excellence (NICE) 2015) and 2) current WHO and International Association of Diabetes and Pregnancy Study Groups (IADPSG) criteria (either fasting ≥5.1 mmol/l or two hour post-load glucose ≥8.5 mmol/l) (Metzger, Gabbe et al. 2010).
(3) In the analyses involving antenatal CMD, the binary GDM variable was replaced with a three-category variable of 1) no GDM, 2) GDM not treated with insulin and 3) GDM treated with insulin.
(4) Prescription codes were removed from both the preconception and antenatal CMD variables and CMD classification based solely on Read codes to investigate any potential misclassification bias (Thielen et al., 2009).

## 3 Results

The demographics of BiB are broadly representative of Bradford as a whole (Wright et al., 2013). Characteristics of the sample and the proportion of missing data in each variable are presented in Table 1. This data stratified by GDM status is also available in supplementary material (S2). 45% of the pregnancies with data on ethnicity were from women of Pakistani ethnicity and mean maternal age was 27 years (standard deviation (SD) 5.6). 26% of the pregnancies with data on education were from women with education higher than A level. Mean booking BMI was 26 kg/m^2^ (SD 5.7). 155 of the pregnancies were non-singleton, 763 pregnancies were affected by pre-eclampsia and/or gestational hypertension and 1028 by GDM (8% of pregnancies with known GDM status). 16% of the pregnancies with data on smoking status were from women who reported tobacco use at some point during pregnancy. 17% of the pregnancies with available data were from women with an indicator of preconception CMD and 11% with an indicator of antenatal CMD.

**Table 1:** Characteristics of the sample (*N*=13,539 pregnancies).

	**n**	**%**
**Ethnicity**		
Pakistani	5071	37.5
White British	4425	32.7
Other	1725	12.7
Missing	2318	17.1
**Maternal age (years)**		
Mean (SD)	27.31 (5.6)	
Missing	1345	9.9
**Multiple pregnancy**		
Singleton	13004	96.1
Multiple pregnancy (twins or triplets)	155	1.1
Missing	380	2.8
**Maternal education**		
Less than 5 GCSE equivalents	2413	17.8
5 GCSE equivalents	3451	25.5
A level equivalents	1622	12.0
Higher than A level	2870	21.2
Other	859	6.3
Missing	2324	17.2
**Pre-pregnancy BMI**		
Mean (SD)	26.03 (5.7)	
Missing	3167	23.4
**Obstetric complication (pre-eclampsia and/or gestational hypertension)**		
Yes	763	5.6
No	11820	87.3
Missing	956	7.1
**Maternal tobacco smoking in pregnancy**		
Yes	1847	13.6
No	9377	69.3
Missing	2315	17.1
**Preconception CMD**		
Yes	2119	15.7
No	10099	74.6
Missing	1321	9.8
**Antenatal CMD**		
Yes	1423	10.5
No	11916	88.0
Missing	200	1.5
**GDM**		
Yes	1028	7.6
No	12044	89.0
Missing	467	3.5

### 3.1 The association between preconception CMD and GDM

#### 3.1.1 Primary analyses

Table 2 displays the results of the association between the exposure of preconception CMD and the outcome of GDM using imputed data. There was some evidence of an effect of preconception CMD on reduced risk for GDM in the unadjusted model. However, this risk was attenuated in the adjusted model. Results of complete case analyses mirrored those of the imputed results; see supplementary material (S3). There was no evidence of an interaction between preconception CMD and ethnicity on risk for GDM (p=0.12 for the interaction term of preconception CMD and ethnicity).

**Table 2:** Associations between preconception CMD and GDM (*N*=13,539 pregnancies).

	GDM			
Unadjusted	%	RR	(95% CI)	p-value
*Preconception CMD* Reference category= no indicator	8.2	1.00		
Preconception CMD indicator	6.4	0.78	(0.65, 0.94)	0.007
Adjusted*	**%**	**RR**	**(95% CI)**	**p-value**
*Preconception CMD* Reference category= no indicator	8.2	1.00		
Preconception CMD indicator	6.4	0.96	(0.80, 1.15)	0.658

Models using Poisson regression within a generalised estimating equation framework with robust standard errors

⁎ adjusted for maternal age, education, ethnicity and obstetric complications of pre-eclampsia and/or gestational hypertension.

#### 3.1.2 Subgroup analyses

There was also little evidence of an association between preconception CMD and GDM within ethnic groups (adjusted RR for women of Pakistani ethnicity 0.92; 95% CI 0.70,1.21 and adjusted RR for women of White British ethnicity 1.05; 95% CI 0.78,1.41), although the direction of effect differed slightly between ethnic groups. Prevalence of GDM was higher in women of Pakistani than White British ethnic origin but White British were more likely to have an indicator of preconception CMD; see supplementary material (S4).

#### 3.1.3 Sensitivity analyses

The sensitivity analyses provided little evidence of an association between preconception CMD and fasting glucose as a continuous variable (adjusted beta 1.01; 95% CI 0.98,1.04). When GDM was classified according to NICE criteria, the proportion of pregnancies affected by GDM in the imputed data was 9.7% (versus 7.9% in the primary analyses). Despite the increased proportion with GDM, this translated into a largely unchanged effect estimate for the association with preconception CMD (adjusted RR 0.96; 95% CI 0.81,1.13). 13.4% of pregnancies were affected by GDM according to WHO and IADPSG diagnostic criteria, with adjusted RR following exposure to preconception CMD also being similar to that of the primary analyses at 1.08; 95% CI 0.94,1.23. When preconception CMD was classified using only Read codes, with prescriptions excluded, this resulted in little change to the effect estimate from primary analysis (adjusted RR 0.86; 95% CI 0.69,1.08).

### 3.2 The association between GDM and antenatal CMD

#### 3.2.1 Primary analyses

The imputed results of the association between the exposure of GDM and the outcome of antenatal CMD are shown in Table 3. There was no evidence of an association on unadjusted or adjusted analyses. Results of complete case analyses mirrored those of the imputed results; see supplementary material (S3). There was no evidence of an interaction between GDM and ethnicity on risk for antenatal CMD (p=0.41 for the interaction term of GDM and ethnicity).

**Table 3:** Associations between GDM and antenatal CMD (*N*=13,539 pregnancies).

	Antenatal CMD			
Unadjusted	%	OR	(95% CI)	p-value
*GDM* Reference category= no GDM	10.7	1.00		
GDM	10.2	0.95	(0.77, 1.17)	0.632
Adjusted*	**%**	**OR**	**(95% CI)**	**p-value**
*GDM* Reference category= no GDM	10.7	1.00		
GDM	10.2	0.91	(0.73, 1.12)	0.368

Models using logistic regression within a generalised estimating equation framework with robust standard errors

⁎ adjusted for maternal age, education, ethnicity, multiple pregnancy, obstetric complications, preconception CMD, maternal smoking and pre-pregnancy BMI.

#### 3.2.2 Subgroup analyses

There was also little evidence of an association within ethnic groups (adjusted OR for women of Pakistani ethnicity 1.16; 95% CI 0.87,1.55 and adjusted OR for women of White British ethnicity 0.75; 95% CI 0.46,1.22), although the direction of effect differed slightly between ethnic groups. Women of White British ethnic origin were more likely to have an indicator of antenatal CMD (supplementary material S4).

#### 3.2.3 Sensitivity analyses

The sensitivity analyses using fasting glucose as a continuous variable in place of the binary GDM variable produced no evidence of an association with antenatal CMD (adjusted OR 0.99; 95% CI 0.88,1.12), nor was there evidence of an association when GDM was re-classified according to NICE (adjusted OR 0.88; 95% CI 0.71,1.09) or WHO and IADPSG criteria (adjusted OR 1.02; 95% CI 0.85,1.23). Likewise, there was no evidence of a difference between groups when GDM was used as a three-category variable encompassing use of insulin; adjusted OR for antenatal CMD in GDM treated with insulin was 0.93 (95% CI 0.70,1.23) and without insulin was 0.88 (0.64,1.21). When antenatal CMD was classified using only Read codes, this also resulted in little change to the effect estimate from primary analysis (adjusted OR 0.90; 95% CI 0.61,1.31).

## 4 Discussion

### 4.1 Main findings

There was no evidence of an association between primary care indicators of CMD prior to pregnancy (preconception CMD) and GDM and between GDM and CMD during pregnancy (antenatal CMD) in a large, multi-ethnic UK birth cohort. There was little evidence for a difference between ethnicities in the association between GDM and CMD in both the preconception and antenatal periods. Reconceptualising the diagnosis of GDM by using fasting glucose or different diagnostic criteria made little difference to these results.

### 4.2 Comparison with previous findings

Research to date on the relationship between GDM and CMD during pregnancy has yielded mixed results; some studies have found evidence of an association, while others have not (Byrn and Penckofer, 2013; Ross et al., 2016). The smaller number of studies examining the association between CMD prior to pregnancy and subsequent GDM have provided evidence supporting a relationship (Beka et al., 2018; Bowers et al., 2013; Clark et al., 2019). Our own meta-analysis of studies during pregnancy and up to one year postpartum, while finding an increased risk of depression in women with GDM during pregnancy, found substantial heterogeneity between studies measuring both symptoms and diagnoses of depression and anxiety (Wilson et al., 2020). Sources of this heterogeneity include variation in measurement of both GDM and mental disorder and also variation in study designs and populations. Indeed most of the studies, which were observational in their design, we assessed as at high risk of bias in their sampling and/or measurement (Wilson et al., 2020). While in our meta-analysis we conducted a number of sensitivity analyses to try to identify similarities in results within studies of a similar design, for example measurement using diagnostic versus screening tools of mental disorder, no clear trends emerged (Wilson et al., 2020). We discuss below some of the strengths of this particular study in the Born in Bradford (BiB) cohort which may have resulted in more conservative effect estimates than that seen in other studies. Other unique aspects of the Bradford population are its high proportion of women of South Asian ethnicity, in which diabetes (both gestational and Type 2) is more common (Di Cianni et al., 2018; Kampmann et al., 2015) so perhaps the experience of GDM is less troubling in these communities than in other ethnic groups in which diabetes is less prevalent.

Another limitation of previous studies is that they often do not report how GDM was diagnosed or consider how the degree of hyperglycaemia may have impacted results. In this study the glucose results of the OGTT were used to consider whether or not the level of hyperglycaemia may have affected risk. This is an important consideration since there is now some evidence of a relationship between elevated maternal glucose concentrations below that of overt diabetes and adverse outcomes (The HAPO Study Cooperative Research Group, 2008). This has caused some to argue that GDM should be conceptualised as a continuum of glucose intolerance and has also led to widespread debate around the diagnostic criteria for GDM (Koning et al., 2018). While one study has reported a positive correlation between levels of hyperglycaemia and depressive and anxiety symptoms (Gezginç et al., 2013), another study found no difference in glucose tolerance between depressed versus healthy groups (Sit et al., 2014). Indeed in the sensitivity analyses we found no evidence for associations with fasting glucose from the OGTT or GDM treated with insulin versus without. We also found no difference in these results when was GDM re-classified according to various diagnostic criteria. This is an important consideration given the recent debate surrounding diagnostic criteria for GDM (Cundy and Holt, 2017).

### 4.3 Strengths and limitations

The BiB cohort provided data that facilitated in-depth exploration of the relationship between GDM and CMD: the first in a UK population. The comprehensive glucose profiles provided by systematic testing for GDM with the OGTT in the cohort also allowed for consideration of how the degree of hyperglycaemia may be influenced by CMD. The multi-ethnic composition of the cohort also facilitated analysis of possible ethnic differences in risk, which has been relatively neglected in the GDM and CMD literature to date (Wilson et al., 2020). There were no significant differences in the association between GDM and CMD observed in this study on stratification by ethnicity.

However, one of the study's limitations is that there may be underdiagnosis of anxiety and depression in primary care (Cepoiu et al., 2008; John et al., 2016). While the prevalence of CMD from primary care indicators in this study is comparable to that of the general population in both the preconception (Copeland et al., 2011; Moffitt et al., 2010) and antenatal periods (Howard et al., 2014), previous analyses of the BiB cohort using screening tools for mental disorder administered in the cohort have suggested that CMD in women of Pakistani ethnicity is twice as likely to be undiagnosed in primary care as women of White British ethnicity (Prady et al., 2016). Indeed in our analyses we also found that women of Pakistani ethnicity were less likely than White British to have an indicator of CMD recorded by primary care in both the preconception and antenatal periods. Research suggests that this under-diagnosis may be for a number of reasons, including stigma and somatisation of symptoms (Bhui et al., 2001; Bhui et al., 2004).

## Conclusions

This is the first study in the UK to examine the relationship between GDM and CMD, although the findings in this multi-ethnic cohort with high levels of deprivation may not generalise to other populations. The lack of evidence for an association in this population between CMD before and during pregnancy and GDM should provide some reassurance to women with GDM and/or CMD.

## Declaration of Competing Interest

KI has received honoraria for educational lectures from Sanofi, Novo Nordisk, Janssen, and Eli Lilly.

## Appendix Supplementary materials

Image, application 1
