# Supplementary material for: Is there an association between anxiety and depression prior to and during pregnancy and gestational diabetes? An analysis of the Born in Bradford cohort
Source: J Affect Disord. 2020 Nov 1;276:345–50. doi: 10.1016/j.jad.2020.07.019 (PMC7477491; doi:10.1016/j.jad.2020.07.019)
Supplement: Supplementary file 1 [file mmc1.docx]

Supplementary material S1: indicators of common mental disorders (CMD) from primary care records

***Prescriptions***

agomelatine, alprazolam, alventa, alventa xl, angilol, ativan, bonilux, bonilux xl, buspirone hydrochloride, chloralbetaine, chloralhydrate, chloral mixture bp2000, cipralex, cipramil, circadin, citalopram, clomipramine, clomipramine hydrochloride, clonezapam, depefex, depefex xl, diazepam, dosulepin, dosulepin hydrochloride, edronax, efexor, efexor xl, escitalopram, feprapax, fluoxetine, flurazepam, fluvoxamine, fluvoxamine maleate, foraven, foraven xl, gamanil, imipramine, imipramine hydrochloride, isocarboxazid, lofepramine, lomont, loprazolam, lorazepam, lormetazepam, lustral, manerix, marplan, melatonin, meprobamate, mianserin, mianserin hydrochloride, mirtazapine, moclobemide, molipaxin, nardil, nitrazepam, optimax, oxactin, oxazepam, parnate, paroxetine, phenelzine, politid, politid xl, propranolol, propranolol hydrochloride, prothiaden, prozac, ranfaxine, ranfaxine xl, reboxetine, seroxat, sertraline, sonata, stilnoct, surmontil, syprol, temazepam, tifaxin, tifaxin xl, tranylcypromine, trazodone, trazodone hydrochloride, trimipramine, tryptophan, valdoxan, venaxx, venaxx xl, venlafaxine, venlafaxine m/r, vensir, vensir xl, welldorm, winfex, winfex xl, zaleplon, zimovane, zispin, zispinsoltab, zolpidem, zolpidem tartrate, zopiclone*,* allegron, anafranil, anafranil sr, chlordiazepoxide, chlordiazepoxide hydrochloride, clomethiazole, cymbalta, doxepin, duloxetine, rivotril, sinepin, sodium oxybate, triptafen, xyrem, yentreve, amitriptyline, amitriptyline hydrochloride, nortriptyline, promethazine, promethazine hydrochloride

***Read codes***

*Depression:*

1B17., 1B19., 1B1U., 2257., 62T1., E112., E1120, E1121, E1122, E1123, E1125, E1126, E112z, E113., E1130, E1131, E1132, E1135, E1136, E1137, E113z, E118., E11y2, E11z0, E11z1, E11zz, E204., E210., E211., E2110, E2112, E2B.., E2B0., E2B1., Eu320, Eu321, Eu322, Eu324, Eu325, Eu326, Eu327, Eu32B, Eu32y, Eu32z, Eu330, Eu331, Eu33y, Eu33z, Eu34., Eu340, Eu34y, Eu34z, Eu3y., Eu3y1, Eu3yy, Eu3z., Eu53., Eu530, X00SO, X00SR, X00SS, X00SU, X00TX, X40Dl, X40Dm, X760u, X7617, X761I, X761J, X761K, X761L, XE0re, XE0uv, XE1Xy, XE1Y0,

XE1Y1, XE1YC, XE1ZY, XE1Za, XE1Zb, XE1Zc, XE1Zd, XE1Zf, XE1Zg, XE1Zh, XE1Zi, XE1aY, XE1ae, XM0Ar, XM0CR, XM1GC, XSEGJ, XSGok, XSGol, XSGom, Xa02E, Xa0wV, Xa110, Xa17z, Xa1eL, Xa9E0, Xa9J0, Xa9K0, XaCHr, XaCHs, XaCIs, XaCIt, XaCIu, XaImU, XaJWh, XaKUk, XaPKm, XaPOv, XaX0C, XaY2C, XaAyL, XaB5v, XaB95, XaB9J

*Anxiety:*

1B13., 1B1V., 2258., 225J., E0300, E0310, E200., E2000, E2001, E2002, E2004, E2005, E200z, E201., E2010, E2011, E2012, E2013, E2014, E2015, E2016, E2017, E2018, E201A, E201B, E201C, E201z, E202., E2020, E2021, E2022, E2023, E2024, E2025, E2026, E2027, E2028, E2029, E202A, E202B, E202C, E202D, E202E, E202z, E203., E2030, E2031, E203z, E205., E207., E20y., E20y0, E20y1, E20y2, E20y3, E20yz, E20z., E28.., E280., E281., E282.,

E283., E2830, E2831, E283z, E284., E28z., Eu40., Eu400, Eu401, Eu402, Eu40y, Eu40z, Eu41., Eu410, Eu411, Eu41y, Eu41z, Eu42., Eu420, Eu421, Eu422, Eu42y, Eu42z, Eu515, Eu51y, Eu51z, Ub1T9, X00Sc, X00Sf, X761N, XE0rb, XE1Y7, XE1YA, XE1Ym, XE1Yn, XE1Zj, XE1aW, XE1bo, XM1MZ, Xa0XG, Xa0XH, Xa0XI, Xa0XJ, Xa0XK, Xa0XM, Xa0XN, Xa0XO, Xa0XP, Xa0XQ, Xa0XR, Xa0XX, Xa0XY, Xa0Xd, Xa18j, Xa18v, Xa19B, Xa3Xk, Xa3Ys, Xa7kB, XaEFB, XaP8d, XaX55, XaX56, XaX58

*Comorbid depression and anxiety:*

E2003, Eu412, Eu413, X00Sb

*CMD treatment or referral for CMD treatment:*

6655., 6659., 66590, 6779., 6G00., 8BK0., 8BM0., 8CQ.., 8CR7., 8F85., 8G..., 8G1.., 8G10., 8G100, 8G11., 8G12., 8G120, 8G121, 8G2.., 8G21., 8G2Z., 8G4.., 8G43., 8G4Z., 8G5.., 8G51., 8G5Z., 8G6.., 8G6Z., 8G7.., 8G7Z., 8G9.., 8G91., 8G9Z., 8HlB., 8HVO., 8H23., 8H230, 8H34., 8H38., 8H49., 8H7A., 8H7B., 8H7T., 8H7Z., 8HHp., 8HHq., 8HJ3., 8HK9., 8HkK., 8HM9., 9HZ.., 9N0T., 9N1M., 9N2B., 9N6h., 9NJ1., 9NJR., 9NJT., 9Ol.., Ub0qs, X71Ec, X71bp, X79sL, XE0iL, XE1Sa, XE1Sb, XSBbs, Xa8IB, Xa8IG, Xa8IJ, Xa8IP, Xa8IR, Xa8If, Xa8Ig, Xa8Ih, Xa8Ii, Xa8Ij, Xa8Ik, Xa8Is, Xa8It, Xa8Iu, Xa8Ix, Xa8J0, XaA8Z, XaA8c, XaA8d, XaA8u, XaA8v, XaA9W, XaA9g, XaABP, XaABQ, XaAKy, XaAMj, XaAMz, XaAOd, XaAOe, XaAOf, XaAOg, XaAOh, XaAQi, XaAQo, XaAS4, XaAU5, XaAUA, XaAXe, XaAZI, XaAbC, XaAbH, XaAdM, XaAel, XaAem, XaAen, XaAfJ, XaAh4, XaAiE, XaAiI, XaAkB, XaAkI, XaAkU, XaAnb, XaBHK, XaBIg, XaBJb, XaBJc, XaBT1, XaBTD, XaBtN, XaBvV, XaBvW, XaBvX, XaCFD, XaECG, XaEVq, XaI8j, XaINQ, XaINy, XaIOf, XaIOg, XaIOh, XaIOi, XaIOj, XaIOk, XaIOl, XaIOn, XaIOp, XaIOq, XaIOs, XaIOu, XaIOv, XaIOy, XaIOz, XaIP0, XaIP1, XaIP2, XaIP3, XaIPw, XaISp, XaISv, XaISw, XaISy, XaIT1, XaIT2, XaIT3, XaIT4, XaIT5, XaIT6, XaIT7, XaIT8, XaITA, XaITG, XaITH, XaITI, XaIUv, XaIUx, XaIUy, XaIUz, XaIV0, XaIV1, XaIV2, XaIV3, XaIV4, XaIV5, XaIV6, XaIW3, XaIW4, XaIW5, XaIW6, XaIWD, XaIWM, XaIWN, XaIWR, XaIWS, XaIWT, XaIWU, XaIWV, XaIWW, XaIWX, XaIWY, XaIWZ, XaIWa, XaIWb, XaIWx, XaIWy, XaIWz, XaIX0, XaIXS, XaIXT, XaIXU, XaIXV, XaIXW, XaIXX, XaIXY, XaIXZ, XaIXa, XaIXb, XaIXh, XaIXi, XaIXk, XaIXl, XaIXm, XaIXn, XaIXo, XaIXp, XaIXq, XaIXs, XaIXt, XaIXu, XaIYN, XaIkd, XaIkg, XaIku, XaIm4, XaIpA, XaItc, XaItx, XaIuR, XaIvk, XaIvp, XaIvq, XaIyU, XaJ4V, XaJ4w, XaJ4x, XaJOA, XaJON, XaJPu, XaJPz, XaJQ1, XaJQD, XaJQE, XaJQF, XaJQG, XaJQH, XaJQI, XaJQJ, XaJQR, XaJQS, XaJQT, XaJQU, XaJQV, XaJQW, XaJQX, XaJQY, XaJQZ, XaJRr, XaJWg, XaJr3, XaK1f, XaK5q, XaK5r, XaK6K, XaK70, XaK71, XaKAx,

XaKEz, XaKGq, XaKbb, XaL03, XaL0o, XaL0p, XaL0q, XaL0r, XaL0s, XaL0t, XaL0u, XaL0v, XaL0w, XaL2L, XaLBl, XaLCP, XaLCQ, XaLFL, XaLFk, XaLNF, XaLQw, XaLnp, XaLnq, XaLnr, XaLst, XaLsu, XaLsv, XaM2K, XaM7s, XaMGz, XaMJ8, XaMhM, XaN3a, XaN4b, XaN4c, XaN4d, XaN4e, XaN4f, XaN4g, XaNPL, XaNTc, XaONq, XaOOT, XaObo, XaOxM, XaP6T, XaP7x, XaPRF, XaPTT, XaPTU, XaPlZ, XaPvy, XaPvw, XaQBz, XaQC0, XaQWJ, XaQvz, XaR4n, XaR4s, XaR5D, XaWzW, XaX04, XaXEJ, XaXH8, XaXHm, XaXe3, XaXiH, XaXl2, XaY6o, XaY7i, XaYgS, XaZIW, XaZcf, ZV663, ZV673, ZV69., ZV690, ZV691, ZV692, ZV6D., ZV701, ZV702

*CMD-related follow-up:*

665.., 6654., 6658., 66580, 665A., 665A0, 665Z., 8A2.., 8A21., 8A2Z., 9H90., 9H91., 9H92., 9HA0., 9Ov.., 9Ov0., 9Ov1., 9Ov2., 9Ov3., 9Ov4., X74WN, XaJuG, XaJuK, XaJuT, XaJuV, XaJuW, XaK6d, XaK6e, XaK6f, XaK9p, XaKAK, XaLIb, XaMGL, XaMGN, XaMGO, XaMGP, XaMGQ, XaMGR, XaR9y, XaZ2p

*CMD History:*

146.., 1465., 1466., 1467., 146A., 146G., 146Z., 9HA1., Eu334, Xa41K, XaJWi, XaLG., ZV111

Supplementary material S2: Characteristics of the sample stratified by GDM status

| **Characteristics of the sample stratified by GDM status** | | | | | |
| --- | --- | --- | --- | --- | --- |
|  | **GDM** | | **No GDM** | | **p value*** |
|  | **N=1028 pregnancies** | **%** | **N=12,044 pregnancies** | **%** |  |
| **Ethnicity** |  |  |  |  |  |
| Pakistani | 536 | 61.0 | 4419 | 44.2 | <0.001 |
| White British | 207 | 23.6 | 4088 | 40.9 |  |
| Other | 136 | 15.5 | 1488 | 14.9 |  |
| **Maternal age (years)** |  |  |  |  |  |
| Mean (SD) | 30.35 (5.5) |  | 27.04 (5.5) |  | <0.001 |
| **Multiple pregnancy** |  |  |  |  |  |
| Singleton | 1006 | 97.9 | 11,911 | 98.9 | 0.003 |
| Multiple pregnancy (twins or triplets) | 22 | 2.1 | 133 | 1.1 |  |
| **Maternal education** |  |  |  |  |  |
| Less than 5 GCSE equivalents | 227 | 25.9 | 2123 | 21.3 | <0.001 |
| 5 GCSE equivalents | 246 | 28.0 | 3108 | 31.1 |  |
| A level equivalents | 95 | 10.8 | 1471 | 14.7 |  |
| Higher than A level | 244 | 27.8 | 2518 | 25.2 |  |
| Other | 66 | 7.5 | 767 | 7.7 |  |
| **Pre-pregnancy BMI** |  |  |  |  |  |
| Mean (SD) | 28.36 (6.1) |  | 25.83 (5.6) |  | <0.001 |
| **Obstetric complication (pre-eclampsia and/or gestational hypertension)** |  |  |  |  |  |
| Yes | 61 | 6.4 | 702 | 6.1 | 0.653 |
| No | 891 | 93.6 | 10909 | 94.0 |  |
| **Maternal tobacco smoking in pregnancy** |  |  |  |  |  |
| Yes | 75 | 8.5 | 1706 | 17.1 | <0.001 |
| No | 806 | 91.5 | 8286 | 82.9 |  |
| **Preconception CMD** |  |  |  |  |  |
| Yes | 135 | 14.4 | 1967 | 17.8 | 0.007 |
| No | 806 | 85.7 | 9082 | 82.2 |  |
| **Antenatal CMD** |  |  |  |  |  |
| Yes | 106 | 10.3 | 1299 | 10.8 | 0.638 |
| No | 922 | 89.7 | 10,745 | 89.2 |  |
|  |  |  |  |  |  |
| **on chi square for categorical variables and t test for continuous variables comparing groups with and without GDM for complete cases with no missing data on exposure* | | | | | |

| Supplementary material S3: Complete case analyses  **Complete case analysis of associations between preconception CMD and GDM (N=9777 pregnancies)** | | | | | |
| --- | --- | --- | --- | --- | --- |
|  | **GDM (N=768 pregnancies)** | | | | |
| **Unadjusted** | **n (%)** | **RR** | **(95% CI)** | | **p-value** |
| *Preconception CMD* Reference category= no indicator | 652 (8.1) | 1.00 |  |  |  |
| Preconception CMD indicator | 116 (6.6) | 0.82 | (0.67, | 0.99) | 0.040 |
|  | | | | | |
| **Adjusted*** | **n (%)** | **RR** | **(95% CI)** | | **p-value** |
| *Preconception CMD* Reference category= no indicator | 652 (8.1) | 1.00 |  |  |  |
| Preconception CMD indicator | 116 (6.6) | 1.01 | (0.84, | 1.23) | 0.896 |
| *Models using Poisson regression within a generalised estimating equation framework with robust standard errors *adjusted for maternal age, education, ethnicity and obstetric complications of pre-eclampsia and/or gestational hypertension.* | | | | | |

| **Complete case analysis of associations between GDM and antenatal CMD (N=9395 pregnancies)** | | | | | |
| --- | --- | --- | --- | --- | --- |
|  | **Antenatal CMD (N=1007 pregnancies)** | | | | |
|  |  |  |  |  |  |
| **Unadjusted** | **n (%)** | **OR** | **(95% CI)** | | **p-value** |
| *GDM* Reference category= no GDM | 932 (10.7) | 1.00 |  |  |  |
| GDM | 75 (10.3) | 0.95 | (0.74, | 1.22) | 0.694 |
|  | | | | | |
| **Adjusted*** | **n (%)** | **OR** | **(95% CI)** | | **p-value** |
| *GDM* Reference category= no GDM | 932 (10.7) | 1.00 |  |  |  |
| GDM | 75 (10.3) | 0.92 | (0.73, | 1.15) | 0.439 |
| *Models using logistic regression within a generalised estimating equation framework with robust standard errors *adjusted for maternal age, education, ethnicity, multiple pregnancy, obstetric complications, preconception CMD, maternal smoking and pre-pregnancy BMI.* | | | | | |

Supplementary material S4: Associations between ethnicity and GDM and CMD using imputed data

| **Associations between ethnicity and GDM (N=13,539 pregnancies)** | | | | | |
| --- | --- | --- | --- | --- | --- |
|  | **GDM** | | | | |
|  | **%** | **OR** | **(95% CI)** | | **p-value** |
| *Ethnicity* Reference category= White British | 4.7 | 1.00 |  |  |  |
| Pakistani | 10.5 | 2.37 | (2.00, | 2.79) | <0.001 |
| Other | 8.1 | 1.78 | (1.41, | 2.25) | <0.001 |
| *Models using logistic regression within a generalised estimating equation framework with robust standard errors.* | | | | | |
| **Associations between ethnicity and preconception CMD (N=13,539 pregnancies)** | | | | | |
|  | **Preconception CMD** | | | | |
|  | **%** | **OR** | **(95% CI)** | | **p-value** |
| *Ethnicity* Reference category= White British | 26.9 | 1.00 |  |  |  |
| Pakistani | 10.8 | 0.42 | (0.37, | 0.47) | <0.001 |
| Other | 10.2 | 0.39 | (0.33, | 0.46) | <0.001 |
| *Models using logistic regression within a generalised estimating equation framework with robust standard errors.* | | | | | |
| **Associations between ethnicity and antenatal CMD (N=13,539 pregnancies)** | | | | | |
|  | **Antenatal CMD** | | | | |
|  | **%** | **OR** | **(95% CI)** | | **p-value** |
| *Ethnicity* Reference category= White British | 11.7 | 1.00 |  |  |  |
| Pakistani | 10.0 | 0.86 | (0.75, | 0.98) | 0.021 |
| Other | 10.0 | 0.84 | (0.70, | 1.01) | 0.065 |
| *Models using logistic regression within a generalised estimating equation framework with robust standard errors.* | | | | | |
